# Diverse Host Immune Responses of Different Geographical Populations of the Coconut Rhinoceros Beetle to Oryctes Rhinoceros Nudivirus (OrNV) Infection

**DOI:** 10.1128/Spectrum.00686-21

**Published:** 2021-09-15

**Authors:** Kayvan Etebari, Maria Gharuka, Sassan Asgari, Michael J. Furlong

**Affiliations:** a School of Biological Sciences, The University of Queensland, Brisbane, Queensland, Australia; b Research Division, Ministry of Agriculture and Livestock, Honiara, Solomon Islands; University of Sussex

**Keywords:** coconut rhinoceros beetle, CRB, *Oryctes rhinoceros*, Oryctes rhinoceros nudivirus, OrNV, invasive pest, transcriptome, gene expression

## Abstract

Incursions of the coconut rhinoceros beetle (CRB), Oryctes rhinoceros, into different islands in the South Pacific have been detected in recent years. It has been suggested that this range expansion is related to an *O. rhinoceros* haplotype reported to show reduced susceptibility to the well-established classical biocontrol agent, Oryctes rhinoceros nudivirus (OrNV). Our understanding of the genetic characteristics which distinguish the population of *O. rhinoceros* that has recently established in Solomon Islands from other well-established populations across the region is very limited. Here, we hypothesized that the recently established *O. rhinoceros* population should have greater innate immune responses when challenged by OrNV than those of well-established and native *O. rhinoceros* populations. We used the RNA sequencing (RNA-Seq) approach to generate gene expression profiles of midgut tissue from OrNV-infected and noninfected individuals collected in the Solomon Islands (recent incursion), Papua New Guinea and Fiji (previously established), and the Philippines (within the native range). The collections included individuals from each of the three major mitochondrial lineages (CRB-G, CRB-PNG, and CRB-S) known to the region, allowing us to explore the specific responses of each haplotype to infection. Although insects from the Philippines and Solomon Islands that were tested belong to the same mitochondrial lineage (CRB-G), their overall responses to infection were different. The number of differentially expressed genes between OrNV-infected and noninfected wild-caught individuals from the four different locations varied from 148 to 252. Persistent OrNV infection caused a high level of induced antimicrobial activity and immune responses in *O. rhinoceros*, but the direction and magnitude of the responses were population specific. The insects tested from the Solomon Islands displayed extremely high expression of genes which are known to be involved in immune responses (e.g. coleoptericin, cecropin, and serpin). These variations in the host immune system among insects from different geographical regions might be driven by variations in the virulence of OrNV isolates, and this requires further investigation. Overall, our current findings support the importance of immunity in insect pest incursion and an expansion of the pest’s geographic range.

**IMPORTANCE** Oryctes rhinoceros nudivirus (OrNV) is a double-stranded DNA (dsDNA) virus which has been used as a biocontrol agent to suppress coconut rhinoceros beetle (CRB) in the Pacific Islands. Recently a new wave of CRB incursions in Oceania is thought to be related to the presence of low-virulence isolates of OrNV or virus-tolerant haplotypes of beetles (CRB-G). Our comparative analysis of OrNV-infected and noninfected CRBs revealed that specific sets of genes were induced by viral infection in the beetles. This induction was much stronger in beetles collected from the Solomon Islands, a newly invaded country, than in individuals collected from within the beetle’s native range (the Philippines) or from longer-established populations in its exotic range (Fiji and Papua New Guinea [PNG]). Beetles from the Philippines and the Solomon Islands that were tested in this study all belonged to the CRB-G haplotype, but the country-specific responses of the beetles to OrNV infection were different.

## INTRODUCTION

Identification of an invasive population and the ability to distinguish invasive individuals from noninvasive individuals in a mixed population are critical steps in pest management. Incursions of the coconut rhinoceros beetle (CRB), Oryctes rhinoceros, into regions that were previously free of the pest have been detected in the South Pacific in recent years (1, 2). This pest was detected in the Solomon Islands for the first time in 2015 (3) and in Vanuatu in 2019 (1). It has been suggested that this range expansion is related to an *O. rhinoceros* haplotype (CRB-G) that has been reported to show reduced susceptibility to the well-established classical biocontrol agent, Oryctes rhinoceros nudivirus (OrNV). This virus was isolated from Malaysian populations of *O. rhinoceros* in the early 1960s and was successfully introduced into the South Pacific islands and kept the coconut rhinoceros beetle at bay for several decades (4).

The CRB-G haplotype is identified by patterns of single nucleotide polymorphisms (SNPs) in the cytochrome *c* oxidase subunit I (*CoxI*) mitochondrial gene (2). However, our recent studies show that the current *CoxI*-based haplotype diagnostics likely do not correlate with any significant biological differences between these insects, and they are only a diagnostic for beetles of a given haplotype lineage that have moved into a region (1). We found three major *O. rhinoceros* mitochondrial lineages (CRB-G, CRB-PNG, and CRB-S) across the South Pacific, and haplotype diversity varied between and within the countries. The *O. rhinoceros* population in most countries is monotypic, but several haplotypes have been reported from a number of countries, including the Solomon Islands, and Palau (1, 5). In these places, where multiple haplotype groups are present and there is obvious introgression between them, the utility of *CoxI*-based haplotype diagnostics is limited, as insects can only be traced back through the maternal line (1, 5).

Resistance to OrNV in the CRB-G mitochondrial lineage has been proposed because of an absence of OrNV infection in wild-caught insects from this haplotype group and the results from laboratory bioassays investigating the susceptibility of adult *O. rhinoceros* from the Guam population of this lineage to orally administered OrNV (2). However, our recent studies demonstrate the high incidence of virus in wild-caught *O. rhinoceros* across the region regardless of their mitochondrial lineage (1) and detection of OrNV in the CRB-G mitochondrial lineage in the Solomon Islands (6), the Philippines (7), and Palau (8). Interestingly, harmonious topologies of both host and OrNV strains provide evidence of codivergence of OrNV strains with their *O. rhinoceros* hosts across the Pacific (7). It has been suggested that *O. rhinoceros* belonging to the CRB-G lineage causes more damage to host palms than insects from the other lineages (9), but definitive evidence is lacking.

Recent incursion of non-CRB-G beetles into Vanuatu (2019) demonstrates the potential of other mitochondrial lineages of *O. rhinoceros* to extend their ranges across the region (1). Not all introduced species become invasive; the establishment and subsequent spread of new arrivals at a given location are determined by the specific genetic characteristics of the introduced individuals and the selective pressures that they encounter in their new environment (10). We still know very little about the genetic characteristics that might distinguish the recently introduced population of *O. rhinoceros* in the Solomon Islands from other previously established populations across the region. We do not know how this potential genetic variation is distributed across the different haplotypes in the region or how the demonstrated admixture between these haplotypes in the Solomon Islands (1) has affected the genetic structure of the *O. rhinoceros* population across the archipelago.

Genetic variation in different geographical populations of *O. rhinoceros* with respect to immune responses to OrNV and genetic variation or virulence of different isolates of OrNV largely determine the outcome of this host-pathogen interaction. Immunity can be core to the success of invasive organisms (11), and as such, we can provide better long-term management strategies for *O. rhinoceros* if we better understand the mechanisms underpinning its responses to this very well-established biological control agent. Here, we hypothesize that the *O. rhinoceros* population in a newly invaded country should have a greater innate immune response to OrNV infection than *O. rhinoceros* populations with longer intimate relationships with the pathogen. Previous studies of the invasive ladybird Harmonia axyridis support a role of immunity in incursion and suggest that population-specific antimicrobial peptide (AMP) gene expression patterns are dynamic and may change more rapidly across different populations than previously thought (12, 13). Immune-challenged invasive populations were shown to induce high expression of genes for several AMPs (e.g., coleoptericins and defensin) compared to the native populations (13). In another study, of invasive Drosophila suzukii, high hemocyte load was associated with increased parasitoid resistance, possibly providing a mechanism by which this species can escape from its natural enemies (14). Female parasitoid wasps inject venom and endogenous virus-like particles to suppress host immune responses, but *D. suzukii* constitutively produces up to five times more hemocytes than Drosophila melanogaster, which suffers high levels of parasitism, thereby providing a mechanism to overcome attack (14).

Although we know very little about immunity and invasion biology in insects, several studies of the biology of invasive bird species compared the immune responses of invasive and noninvasive avian hosts exposed to the same pathogen (reviewed in reference 11). For example, invasive house sparrows (Passer domesticus) in North America express greater humoral antibody-mediated responses than their less invasive congener, the Eurasian tree sparrow (Passer montanus) (15). Fassbinder-Orth et al. also reported enhanced immune responses in an invasive bird to Buggy Creek virus (*Togaviridae*: *Alphavirus*) and infestation of its arthropod vector, the swallow bug (Oeciacus vicarius), compared to the native swallow (Petrochelidon pyrrhonota) (16).

In this study, we concentrated on the immune responses of wild-caught *O. rhinoceros* to viral infection in the context of how long they have been established in a region. We used the RNA sequencing (RNA-Seq) approach to generate gene expression profiles of the midgut from OrNV-infected and uninfected *O. rhinoceros* individuals from four different geographical populations representing the native range, a region where they have been long established, and a region that has been invaded more recently. We included all three major mitochondrial lineages (CRB-G, CRB-PNG, and CRB-S) in the study to explore the specific responses of beetles from each lineage to OrNV infection. Our findings support the importance of immunity in insect pest incursion and an expansion of the pest’s geographic range, as suggested by Lee and Klasing (17) and experimentally demonstrated in other insects, such as *H. axyridis* (13, 18). The study also expands our understanding of *O. rhinoceros* gene expression profiles and their transcript sequences, which will facilitate future studies that could determine an appropriate biomarker to distinguish between invasive and noninvasive populations.

## RESULTS AND DISCUSSION

### RNA-Seq data analysis and gene expression profile.

As midgut tissue is the most active site for viral entry and replication in *O. rhinoceros* (19), the midgut gene expression profiles were generated in response to OrNV infection in four different geographical populations of the beetle. We performed Illumina-based high-throughput sequencing on poly(A)-enriched RNAs extracted from OrNV-infected and uninfected *O. rhinoceros* individuals, and in total, 928,452,708 raw reads were produced from 20 total RNA libraries (Table 1).

**TABLE 1:** The total number of reads in each RNA-Seq library and the samples’ virus infection status and mitochondrial lineage

Name	OrNV status	No. of reads	No. of reads after trim	Mapped to OrNV genome (%)	Haplotype (*Cox*I)
FIJ 1	Infected	52,543,022	51,840,997	0.91	CRB-S
FIJ 2	Infected	40,335,112	39,810,518	6.34	CRB-S
FIJ 3	Uninfected	46,016,340	45,412,371		CRB-S
FIJ 4	Uninfected	47,222,794	46,605,945		CRB-S
FIJ 5	Uninfected	47,325,904	46,692,881		CRB-S
PNG 1	Infected	43,356,384	42,806,613	3.21	CRB-PNG
PNG 2	Infected	43,949,524	43,342,275	12.65	CRB-PNG
PNG 3	Infected	42,510,768	41,923,395	11.87	CRB-PNG
PNG 4	Infected	41,598,826	40,992,462	13.62	CRB-PNG
PNG 5	Uninfected	46,825,700	45,932,217		CRB-PNG
PHI 1	Infected	44,478,956	43,810,718	18.92	CRB-G
PHI 2	Infected	47,597,710	46,922,988	13.22	CRB-G
PHI 3	Infected	58,581,084	57,558,495	0.93	CRB-G
PHI 4	Uninfected	47,264,160	46,606,170		CRB-G
PHI 5	Uninfected	48,006,922	47,366,586		CRB-G
SI 1	Infected	53,068,970	52,309,721	14.33	CRB-G
SI 2	Infected	46,657,322	46,031,625	0.54	CRB-G
SI 3	Uninfected	43,088,672	42,479,378		CRB-G
SI 4	Uninfected	41,769,692	41,264,372		CRB-G
SI 5	Uninfected	46,254,846	45,700,143		CRB-G

Maximum likelihood phylogeny analysis of four different geographical populations of *O. rhinoceros* based on their complete mitochondrial genome sequence consensus generated three major mitochondrial lineages, CRB-G, CRB-S, and CRB-PNG (1). All *O. rhinoceros* individuals collected from PNG (Kimbe, New Britain) and Fiji belonged to the mitochondrial lineages CRB-PNG and CRB-S, respectively. The individuals from the Philippines, which is within the native range of *O. rhinoceros*, and the recently invaded Solomon Islands belonged to the CRB-G lineage (see Fig. S1 in the supplemental material).

*De novo* assembly of sequenced data generated 47,056 contigs between 500 and 22,592 bp in length with *N*_25_, *N*_50_, and *N*_75_ values of 2,681, 1,535, and 908 bp, respectably. *N*_50_ is a parameter to show the quality of assembly; it measures the continuity of contigs and is defined as the sequence length of the shortest contig at 50% of the total length of the assembled transcriptome (20). A similarity search revealed that almost 55% (25,812) of these sequences did not produce any hits against the NCBI protein database (nonredundant [nr]) in BLASTx. The closely related scarab beetle, Oryctes borbonicus, the taurus scarab (Onthophagus taurus), red flour beetle (Tribolium castaneum), and the Asian long-horned beetle (Anoplophora glabripennis) were the top hit species for BLAST. Around 28% of total sequences (12,955) have been annotated with different levels of Gene Ontology (GO) terms. A list of the top 200 highly expressed sequences, representing mostly housekeeping genes and ribosomal proteins, is summarized in Table S1. As expected, and previously shown (21), a small number of assembled contigs belong to gut bacterial flora, and these were manually removed from the list.

### Differential gene expression profile in response to OrNV infection.

**FIG 1:**
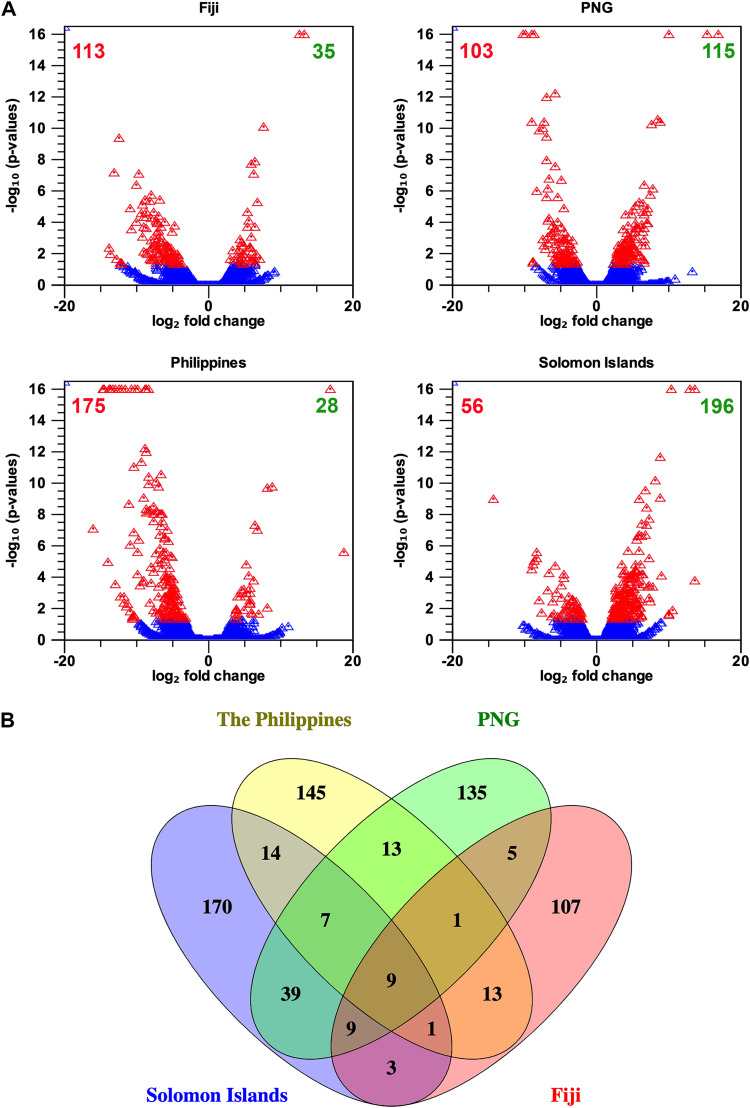
The number of differentially expressed genes (DEGs) in each geographical population of *O. rhinoceros*. (A) Volcano plot analysis. Red triangles indicate differentially expressed transcripts in response to OrNV infection (log_2_ fold change, >2; FDR, <0.05). The numbers in red and green are the numbers of downregulated and upregulated sequences in each population, respectively. (B) The number of differentially expressed genes (DEGs) varied between 148 to 252 among four different geographical populations when we compared their OrNV-infected and noninfected individuals. Both populations from the Philippines and Solomon Islands are from the CRB-G haplotype, but their overall response to infection is different. There are only 31 common DEGs when comparing the altered genes between these two populations, while 64 common DEGs were found between Solomon Island and PNG populations.

Analysis and comparison of gene expression profiles of OrNV-infected and uninfected adult *O. rhinoceros* beetles revealed significant modulation (log_2_ fold change, ≥2; false-discovery rate [FDR] *P* value, ≤0.05) of 886 contigs, regardless of the geographical location from which they were collected (Table S2). The number of differentially expressed genes (DEGs) varied from 148 to 252 among four different geographical populations when OrNV-infected and OrNV-free individuals were compared (Table S3). The maximum number of DEGs was identified in the Solomon Islands population, while the lowest number of DEGs was recorded in individuals collected from Fiji (Fig. 1A). In insects from PNG and the Philippines, OrNV infection affected the expression of 218 and 203 sequences, respectively, but only 30 of these DEGs were common to insects from both locations (Fig. 1 and Table S3).

Depletion of altered genes was a notable pattern in OrNV-infected beetles from Fiji and the Philippines. Although the populations from the Philippines and Solomon Islands consist of individuals from the CRB-G mitochondrial lineage, their overall responses to infection were very different (Fig. 1 and Table S3). We identified only 31 common DEGs when comparing the altered genes between these two populations, while 64 common DEGs were found between Solomon Island and PNG populations, demonstrating the similarity between individuals from these two populations (Fig. 1B). We recently used genotyping-by-sequencing to study *O. rhinoceros* population genetics across the Pacific, and our data provided evidence for gene flow and admixture between different mitochondrial haplotypes in the Solomon Islands (1). Data based on nuclear DNA samples and the STRUCTURE analysis placed insects from PNG and the Solomon Islands together but separate from beetles from Fiji (1) The similarity of the gene expression profile of *O. rhinoceros* in response to OrNV infection reported here is consistent with the previous study (1) and provides another layer of evidence supporting less genetic diversity between these two populations regardless of their mitochondrial haplotypes.

Interestingly, the strains of OrNV present in PNG and Solomon Islands are noticeably different, while the virus found in individuals in the Solomon Islands is closely related to the Philippines isolate (7). In a previous study, we detected several polymorphic sites in 892 positions of the viral genome among different geographical populations. Nonsynonymous mutations were also detected in several OrNV hypothetical proteins and 15 nudivirus core genes, such as *gp034*, *lef-8*, *lef-4*, and *vp91*. We suggested that some of these genomic changes, which are specific to the geographic population, could be related to particular phenotypic characteristics of the strain, such as viral pathogenicity or transmissibility, and this requires further investigation (7). Therefore, to some extent, the overall changes in the host immune response to OrNV infection in these geographical populations could be due to the diverse responses to different strains of the virus that infect these populations. We should expect to see a continuous coevolutionary process involving both host immune response and viral escape mechanisms in this host-virus interaction because OrNV has been well established in *O. rhinoceros* populations in the Pacific region for many decades. It has been demonstrated that viruses which infect a single host lineage would be subjected to specific host-induced pressures and, therefore, be selected by them (22). In a place like the Solomon Islands, where there are several host lineages, the situation is more complex for both the virus and its host. In the rest of this article, we will focus on differentially expressed genes related to the host immune responses to the viral infection.

Gene Ontology (GO) analysis showed that the majority of DEGs participated in cellular and metabolic processes and biological regulation. Binding and catalytic activity were the most abundant GO terms (at level 2) for biological function. The DEGs in insects from the Philippines had the lowest level of GO term enrichment for biological function. There was no difference in the GO term enrichment for cellular components between any of the geographical populations. Cellular component GO term distribution shows virus infection-modified genes which are involved in cellular anatomical entity, intracellular, and protein-containing complexes (Fig. S2).

### Impact of OrNV infection on pattern recognition receptors.

**FIG 2:**
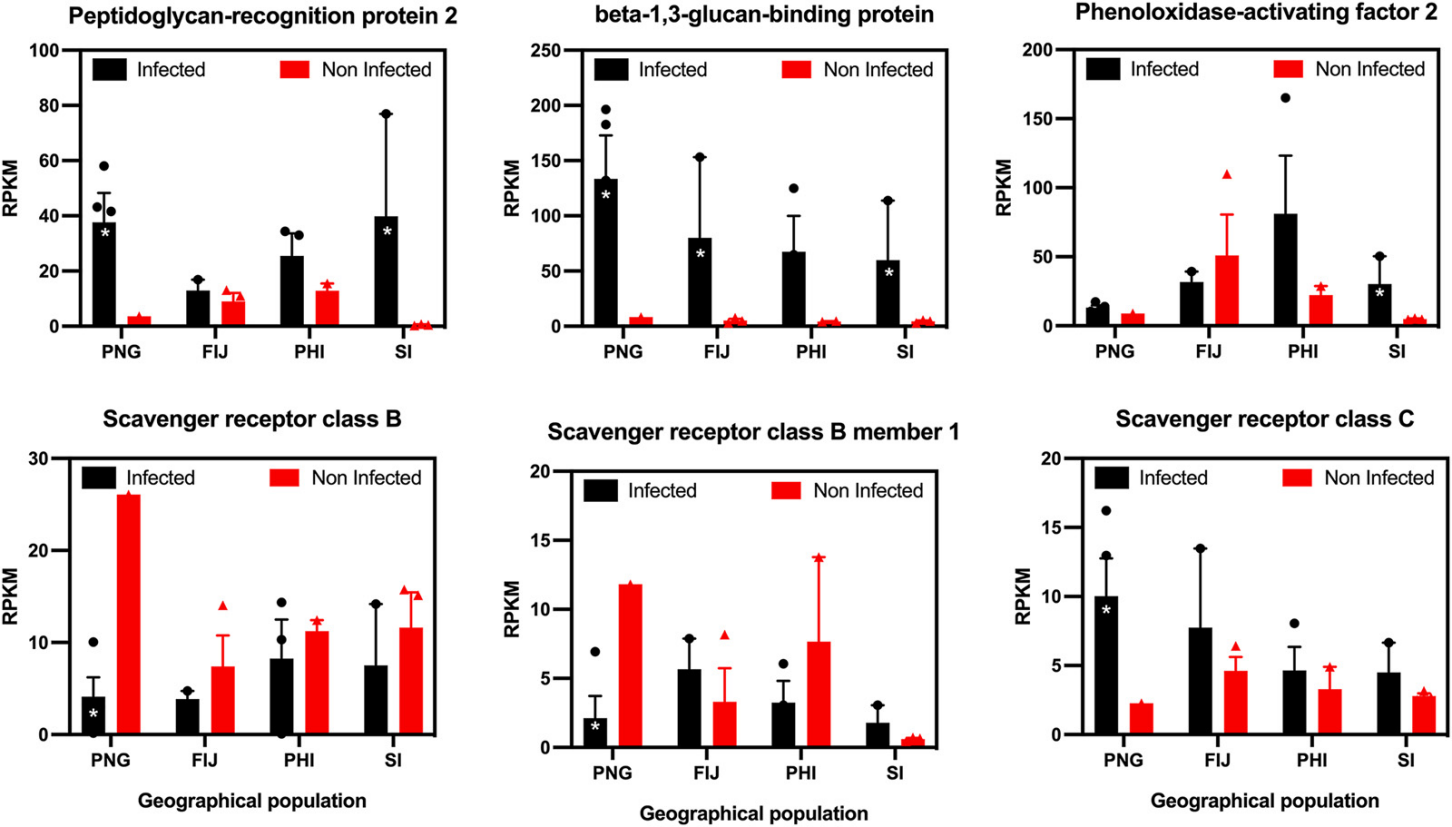
The expression values of some *O. rhinoceros* genes involved in immune response and pathogen recognition pathways. Significant induction was observed in pattern recognition transcripts in OrNV-infected beetles (top row). Scavenger receptor showed various alterations in different populations (bottom row). Genes were considered significantly modulated (shown with an asterisk) if the fold change was >2 and the FDR *P* values were <0.05.

The first step of an immune response is the detection of invading microbes when they enter the insect body. Peptidoglycan recognition proteins (PGRPs) are the most important pattern recognition receptors (PRRs) in insects and other arthropods (23). We identified several contigs which showed homology to PGRPs in *O. rhinoceros* transcriptome data. Overall, induction of PGRP-2 (contig 9863) in OrNV-infected individuals was detected in all geographical populations, but overexpression was statistically significant in Solomon Island (log_2_ fold change [FC], 5.9; FDR, <0.0001) and PNG (log_2_ FC, 3.13; FDR, 0.04) populations (Fig. 2).

Overexpression of an *O. rhinoceros* gene which encodes beta-1,3-glucan binding protein was also detected in all the geographical populations in response to OrNV infection (contig 10148, log_2_ FC, >3). Upregulation of beta-1,3-glucan binding protein in white spot syndrome virus (WSSV)-infected shrimp, Penaeus stylirostris, which activates the prophenoloxidase (proPO) cascade, was previously reported (24). WSSV is a large double-stranded DNA (dsDNA) virus of an arthropod-infecting virus family, *Nimaviridae*, a family closely related to nudiviruses (25). This class of proteins is associated with fungal infection in insects, and they play critical roles in the activation of the melanization pathways through triggering the proPO cascade. However, recent studies have shown that they are also involved in the recognition of the lysine-type peptidoglycans upstream of the Toll pathway, which enables antimicrobial peptide production in insects (26). Here, we also detected significant overexpression (log_2_ FC, 2.22; FDR, 0.04) of phenoloxidase-activating factor 2 (contig 2867) in OrNV-infected samples from the Solomon Islands (Fig. 2).

Scavenger receptors (SRs) are a group of endocytic PRRs with ligand-binding activity, which directly bind to proteins, polyribonucleotides, polysaccharides, and lipids (27, 28). The role of scavenger receptors in pathogen recognition and innate immunity has been documented in insects and other arthropods (29–32). We only detected statistically significant alteration of SRs in beetles collected from PNG, which showed substantial suppression of two members of class B (contigs 6487 and 9881) and overexpression of a class C ortholog (contig 15405). Noticeable suppression of SR class B member 1 was detected in individuals from PNG and the Philippines. Although both populations in the Solomon Islands and the Philippines belong to the CRB-G mitochondrial lineage, they showed different SR expression patterns in response to OrNV. Nudiviruses, like baculoviruses, herpesviruses, and poxviruses, conceivably inhibit host cellular apoptosis by encoding antiapoptotic proteins to suppress the host immune system. Different classes of SRs are necessary for caspase activation and subsequent apoptosis in macrophage cells (33, 34). As is evident from Fig. 2, suppression of some of these SRs by OrNV is a possible way in which the virus manipulates the host immune response. Viruses have developed a wide range of strategies, such as host gene modulation, to not only avoid host immune detection, but also to actively manipulate immune responses to use essential cellular machinery to replicate and establish their infection (35).

A previous study showed that the endoparasitic wasp Diadegma semiclausum, which injects venom proteins and polydnaviruses into its host, significantly suppresses the transcription levels of two SRs in its Plutella xylostella host larvae (36). Interestingly, the transient expression of two polydnavirus genes (DsIV Vankrin 1 [Vank1] and Repeat element 4) drastically suppressed the expression of these two SRs in *P. xylostella* cell culture (36). The swimming crab (Portunus trituberculatus) SR class B transcripts were also downregulated after challenge with WSSV (37). However, upregulation of *Croquemort* (SR class B), another member of the CD36 superfamily, was reported in lymphoid organs of kuruma shrimp (Marsupenaeus japonicus) at 72 and 120 h postinfection with WSSV (38). The SR gene expressions and function are highly tissue- and stage-specific, because in many cases the posttranslational modifications of the CD36 domain, such as glycosylation, phosphorylation, acylation, and palmitoylation vary in different cell types (39).

We could not find any meaningful correlation between the virus load and SR expression values in infected beetles, but downregulation of one member of class B SRs was recorded in all geographical populations (Fig. 2). Perhaps, SRs play an important role during the course of OrNV infection in *O. rhinoceros*, but we do not know if SRs facilitate virus movement or entry within the insect body or initiate an immune response against OrNV infection. Interestingly, a recent study showed that lack of the scavenger receptor cysteine-rich domain 5 of CD163 in pigs is a cause of resistance to porcine reproductive and respiratory syndrome virus 1 (PRRSV) infection because CD163 acts as a fusion receptor for PRRSV and the interaction site for the virus (40). Further investigation is required to improve our understanding of the role of SRs in the interactions between OrNV and its host.

Peritrophin, a component of the peritrophic matrix, also plays an important role in the midgut and protects insects from toxins and pathogens (41). The overexpression of a sequence, which showed a high degree of similarity to *O. taurus* peritrophin-1-like protein, was detected in all wild-caught OrNV-infected *O. rhinoceros* beetles except individuals from the Philippines (Tables S2 and S3). Although this gene is not highly expressed, its degree of overexpression in individuals from the Solomon Islands (log_2_ FC, 7.19; FDR *P* value, 0.0004) is significantly higher than that in individuals from other locations (Fiji log_2_ FC, 2.63; Fiji FDR *P* value, 0.03; PNG log_2_ FC, 3.98; PNG FDR *P* value, 0.0023). We also identified some mucin-like peritrophin transcripts with serine-type endopeptidase and scavenger receptor activity in the *O. rhinoceros* midgut transcriptome, but their expression levels did not change in response to infection. Binding to Gram-negative bacteria and strong binding activity to chitin indicate the involvement of this protein in the insect immune response. This protein protects insects from invasion of microorganisms and stimulates digestion of food in the midgut.

### Host RNAi response modulated by OrNV infection.

**FIG 3:**
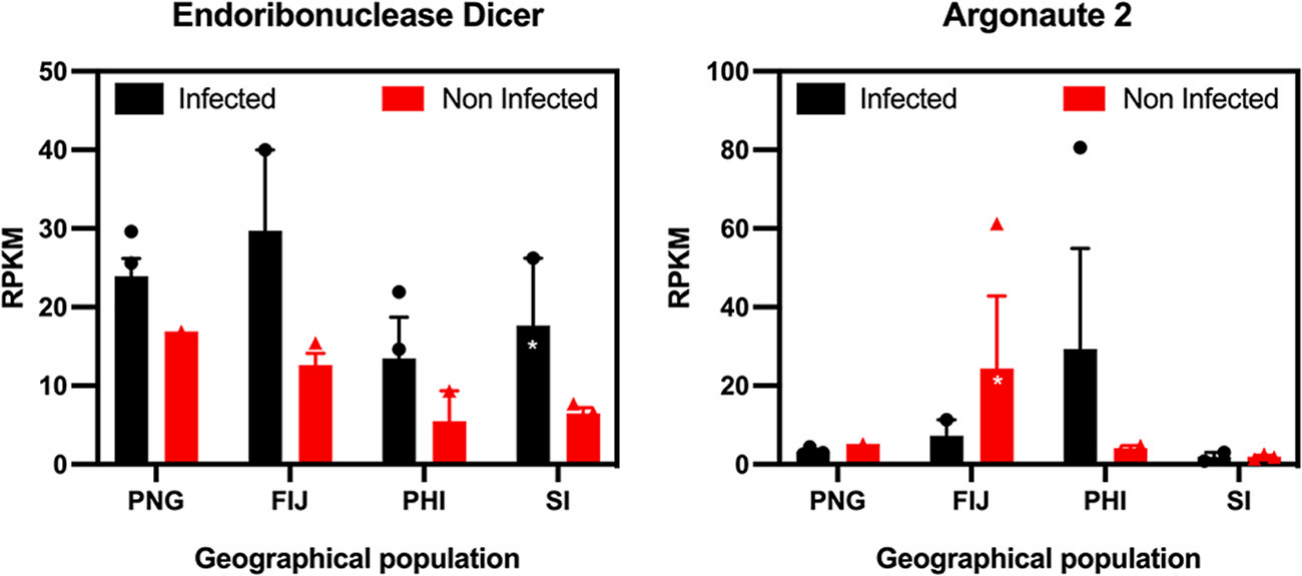
RNA interference (RNAi), which is a major innate immune pathway altered by OrNV infection in *O. rhinoceros*. The expression of a Dicer-like sequence in all infected beetles was upregulated, but this overexpression was only statically significant in samples collected from the Solomon Islands. We did not find any specific pattern of change for Argonaute among infected and noninfected individuals. Significant induction of Ago-2 like sequence upon infection in the Philippine population and significant depletion in wild-caught OrNV-infected individuals from Fiji. Genes were considered significantly modulated (shown with an asterisk) if the fold change was >2 and the FDR *P* values were <0.05.

RNA interference (RNAi) is a major innate immune pathway in insects (42). We checked the expression pattern of the genes involved in the RNAi response in OrNV-infected and uninfected individuals from the different geographical populations. During virus infection in arthropods, two standard small RNA (sRNA) classes, the microRNAs (miRNAs) and small interfering RNAs (siRNAs), contribute to the antiviral response and promote viral tolerance and, in some cases, clearance of virus infections (43, 44). RNAi is initiated by intracellular detection of exogenous long dsRNA. Endoribonuclease Dicer-2 is an enzyme with an RNase motif, which is one of the most important proteins in the recognition of viral dsRNA in the siRNA pathway. The expression of a Dicer-like sequence in all infected beetles was upregulated, but this overexpression was only statically significant in samples collected from the Solomon Islands (Fig. 3). We recently showed that OrNV in the Solomon Island population is targeted by the host RNAi response by mapping small RNA reads to the OrNV genome sequence (7).

Argonaute-2 (Ago2), as an important protein in the siRNA pathway, is part of a multiprotein RNA-induced silencing complex (RISC) that cleaves viral mRNA by its “slicer” endonuclease activity (44). We also checked the expression profile of this gene but could not find any specific pattern of change among infected and noninfected individuals. Our data showed significant induction of Ago-2-like sequence upon infection in beetles from the Philippines and significant depletion in OrNV-infected individuals from Fiji (Fig. 3).

There is growing body of evidence indicating that the RNAi response is not only an important pathway against RNA viruses, but also plays a critical role in the response to DNA viruses (42, 45). Silencing of the RNAi pathway components (i.e., Dicer-2, R2D2, and Ago-2) in some dipteran and lepidopteran cell lines led to an increase in the replication of dsDNA viruses such as *Iridoviridae*, *Poxviridae*, and *Baculoviridae* (46–49).

Recently, Palmer et al. reported the importance of the RNAi-mediated antiviral pathways in D. melanogaster against Kallithea virus (KV; *Nudiviridae*) using mutant fly lines (45). To the best of our knowledge, our study provides the first evidence of the upregulation of a Dicer-like sequence and activation of the RNAi response to a nudivirus infection in a nondipteran insect. It has been shown that some DNA viruses, such as Heliothis virescens, ascovirus-3e (*Ascoviridae*) and invertebrate iridescent virus type 6 (*Iridoviridae*) encode proteins to suppress the host RNAi responses, but there is no evidence for such a gene in members of the family *Nudiviridae* (44, 50).

### OrNV infection induces antimicrobial peptide transcription.

**FIG 4:**
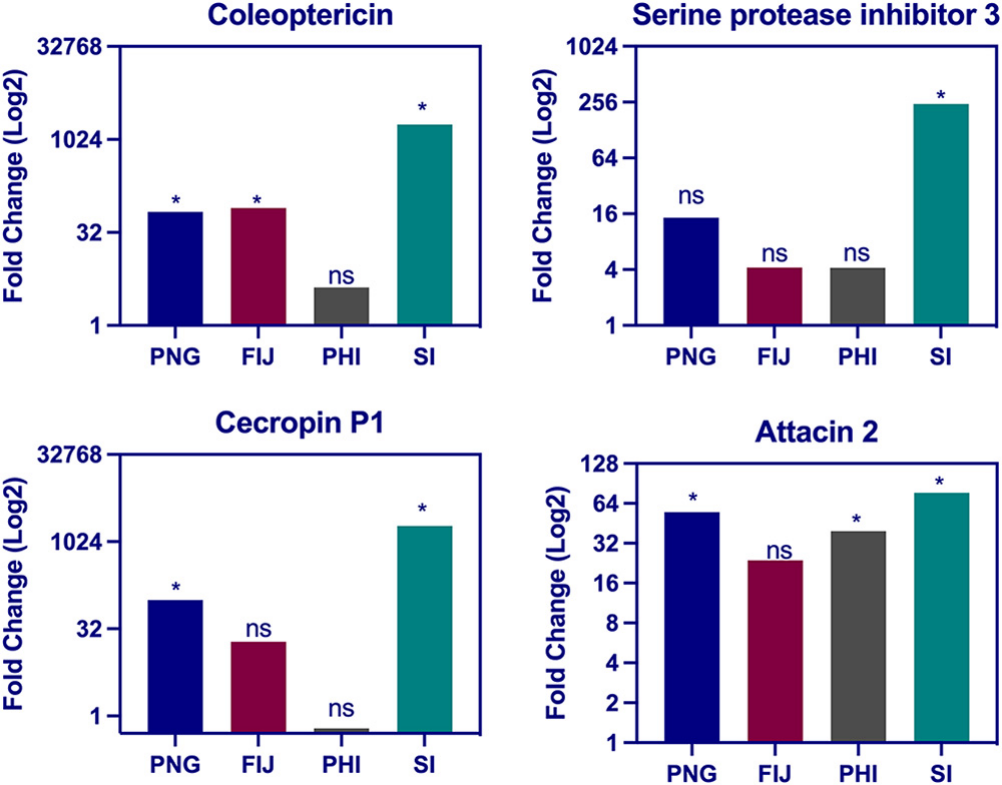
Overexpression of genes which encode antimicrobial peptides was observed in wild-caught OrNV-infected *O. rhinoceros*. The bar charts show the fold change (log_2_) of some selected sequences, and more details are available in Table S3. Genes were considered significantly modulated (shown with an asterisk), if the fold change was >2 and the FDR *P* values were <0.05.

OrNV infection causes a high level of induced antimicrobial activity and immune responses in *O. rhinoceros* beetles, but the direction and magnitude of the response is population-specific. Our data elucidated that those individuals from the recently invaded Solomon Islands display much higher induction of immune-related genes such as antimicrobial peptides (AMPs), lysozymes, and serine protease inhibitors (Serpin) in OrNV-infected samples compared with individuals from the native population in the Philippines or individuals from the populations that have established in Fiji and PNG (Fig. 4 and Table S3).

Coleoptericin domain-containing proteins are a series of important AMPs in Coleoptera (51). Rhinocerosin is a previously characterized coleoptericin domain-containing protein in *O. rhinoceros* (52), and its low expression has been detected in noninfected *O. rhinoceros* larvae and adults (21). We observed upregulation of coleoptericin in response to OrNV infection in all geographical populations (Fig. 4 and Tables S2 and S3), but this overexpression is profound in the recently established population of *O. rhinoceros* in the Solomon Islands (log_2_ FC, >10). Previous studies showed upregulation of this protein in mealworm beetle (Tenebrio molitor) parasitized by Scleroderma guani (53) and after bacterial challenge (54). It has been shown that the expression and production of this AMP is dependent on the immune deficiency (Imd) pathway in the cereal weevil *Sitophilus* (55). These proteins have received more attention since Verheggen et al. suggested that coleoptericins can play a key role in supporting the invasive performance of *H. axyridis* (18).

Cecropin, is another well-known AMP which showed a huge induction (cecropin P1 log_2_ FC, >10) in infected individuals from the Solomon Islands (Fig. 4 and Table S3). A recent study in silkworm, Bombyx mori, screened host AMPs using proteomics and showed upregulation of cecropin D2 and B after infection with Bombyx mori nucleopolyhedrovirus (BmNPV), while cercropin D exhibited a negative regulatory correlation (56). Here, we only identified one sequence with homology to *cecropin* (Cp1) in *O. rhinoceros*, but further investigation is required to characterize other potential AMPs of *O. rhinoceros*.

Overexpression of a sequence with similarity to *attacin* has also been detected in OrNV-infected samples (Fig. 4). The *attacin* expression value of OrNV-infected individuals in the PNG, Philippine, and Solomon Island populations was 44, 30, and 62 times higher than their noninfected group, respectively (Fig. 4 and Tables S2 and S3). Attacins are glycine-rich AMPs which have been reported in a wide range of insects and are known to be active only against growing Gram-negative bacteria (57). However, their overexpression in *T. molitor* in response to infection by the Gram-positive bacterium, Staphylococcus aureus has also been documented (58). A recent study reported the overexpression of *attacin* in BmNPV-infected silkworm and showed that the transcriptional levels of *attacin* progressively increased with time postinfection and were maintained at high levels of expression for an extended period (56). Its highest expression rate among all other differentially expressed AMPs suggested a leading role for attacin protein in humoral immunity against BmNPV in silkworm (56). Significant induction of *attacin* in the fat body of Aedes aegypti after exposure to the entomopathogenic fungus Metarhizium anisopliae has also been reported (59), and it has been suggested the activation of the Toll pathway in response to fungal infection triggers the transcriptional activation of *attacin* in infected mosquitoes.

We identified two sequences (contigs 21087 and 28480) with similarity to Toll protein, which was significantly upregulated (log_2_ FC, >3; FDR, <0.003) due to OrNV infection in *O. rhinoceros* adult beetles (Table S2). However, we could not detect any significant changes in the expression value of this gene within the geographical populations of *O. rhinoceros*. Palmer et al. reported that the Toll pathway mediates antiviral defense against Kallithea virus (KV), which is a member of the *Nudiviridae*, but virus-encoded protein gp83 plays a critical role to suppress host immune response by inhibiting Toll signaling (45). They also showed that *Drosophila* mutants for the RNAi and Imd pathways, but not Toll, are more susceptible to Kallithea virus infection. Previously, we demonstrated differential expression of *OrNV_gp054*, which is a homolog of *gp83*, in infected *O. rhinoceros* beetles among different geographical populations (7). The lowest expression level of this viral gene (*OrNV_gp054*) was detected in the beetles from the Solomon Islands (7). Likely, *OrNV_gp054* plays a role similar to that of *KV_ gp83* and suppresses the *O. rhinoceros* immune response by inhibiting Toll signaling. The low expression level of *OrNV_gp054* in beetles from the Solomon Islands could be a reason for the remarkable induction of AMPs in these individuals. These findings suggest potential interactions between this viral gene and the host humoral immune response.

**FIG 5:**
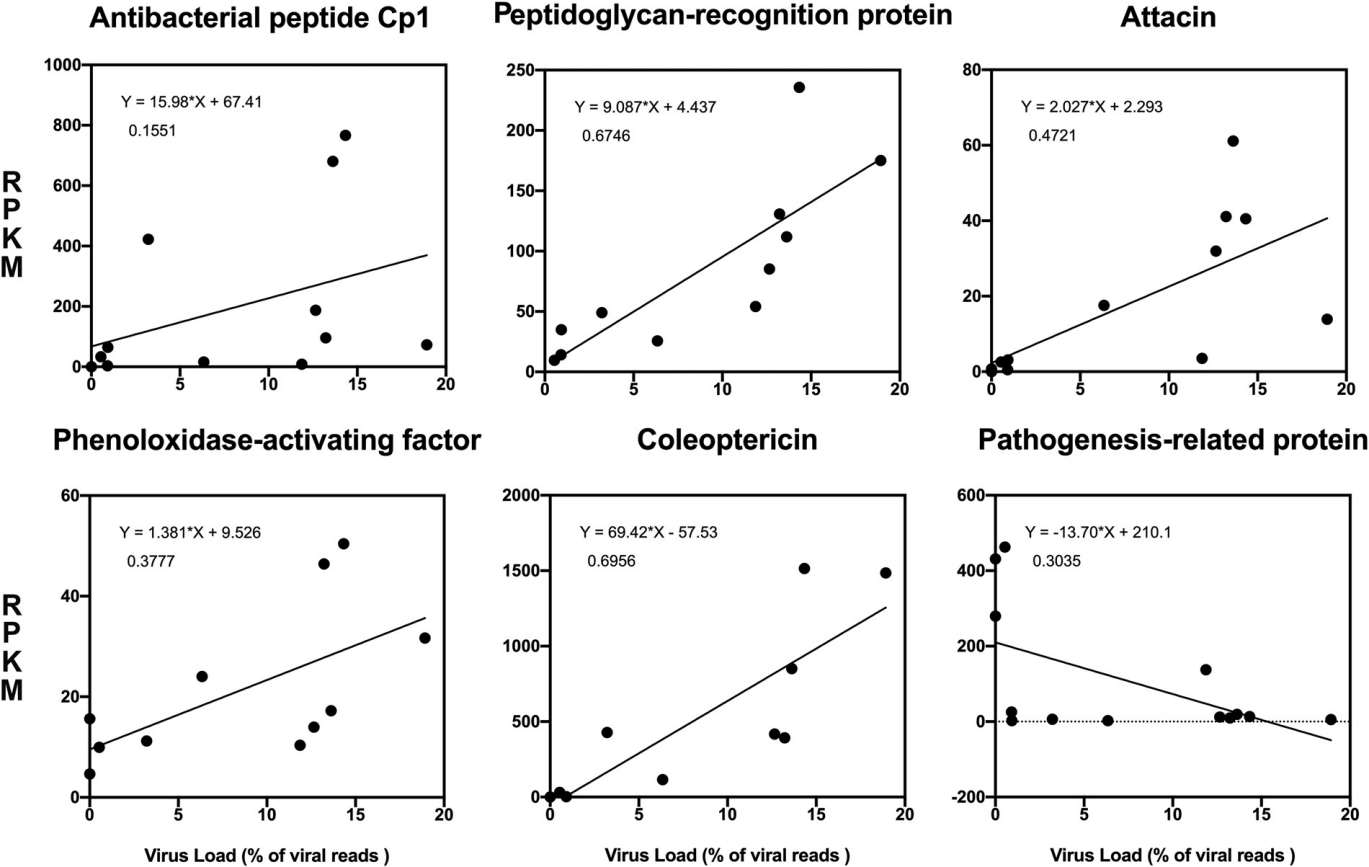
The correlation between virus load and immune-related gene expression values. The total number of reads mapped to the viral genome was used as an indication of viral load in infected beetles.

We used the total number of reads mapped to the viral genome as an indication of viral load in infected beetles and examined their correlation with some AMPs and other dysregulated *O. rhinoceros* genes. Here, we found a positive correlation with the viral load in a few AMPs, such as attacin, cecropin, and coleoptricin, in *O. rhinoceros* (Fig. 5). A negative correlation with viral load was found in a sequence (contig 21261) which showed a high degree of similarity with “pathogenesis related protein” and “thaumatin” in a BLAST search. Thaumatins are another group of AMPs which were primarily discovered as antifungal proteins in plants but were later found in insects, including *O. rhinoceros* (21, 60). Previous studies did not detect the expression of thaumatin in *O. rhinoceros* fat body, and it has been shown that this gene is highly expressed in the midgut tissue (21). This significant downregulation due to OrNV infection (Table S2) and negative correlation with viral load (Fig. 5) suggest potential modulation of this gene by the virus to suppress the host immune response.

As the modulation of these AMPs in response to different kinds of immune challenges such as viruses, bacteria, fungi (reviewed in reference 61), and even parasitization (62) has been demonstrated, Dobson et al. (58) proposed a conjoined mechanism for the expression of AMPs regardless of whether the products are effective against the infection. However, these AMPs may be involved in different immune pathways. For example, a notable increase in BmNPV was observed upon dsRNA-mediated knockdown of seroin in *B. mori.* Analysis of bacterial load upon knockdown of this antiviral protein resulted in higher proliferation of bacteria, which indicates involvement of this protein in several immune pathways (63). Further, previous studies showed the long-lasting immune response to a range of microbial infections in *Tenebrio*, mostly resulting from overexpression of a few AMPs. Makarova et al. explained this AMP activation by a lack of specific pathogen recognition or, alternatively, by synergistic interactions between AMPs (64).

### Comparison of gene expression profiles of noninfected beetles.

**FIG 6:**
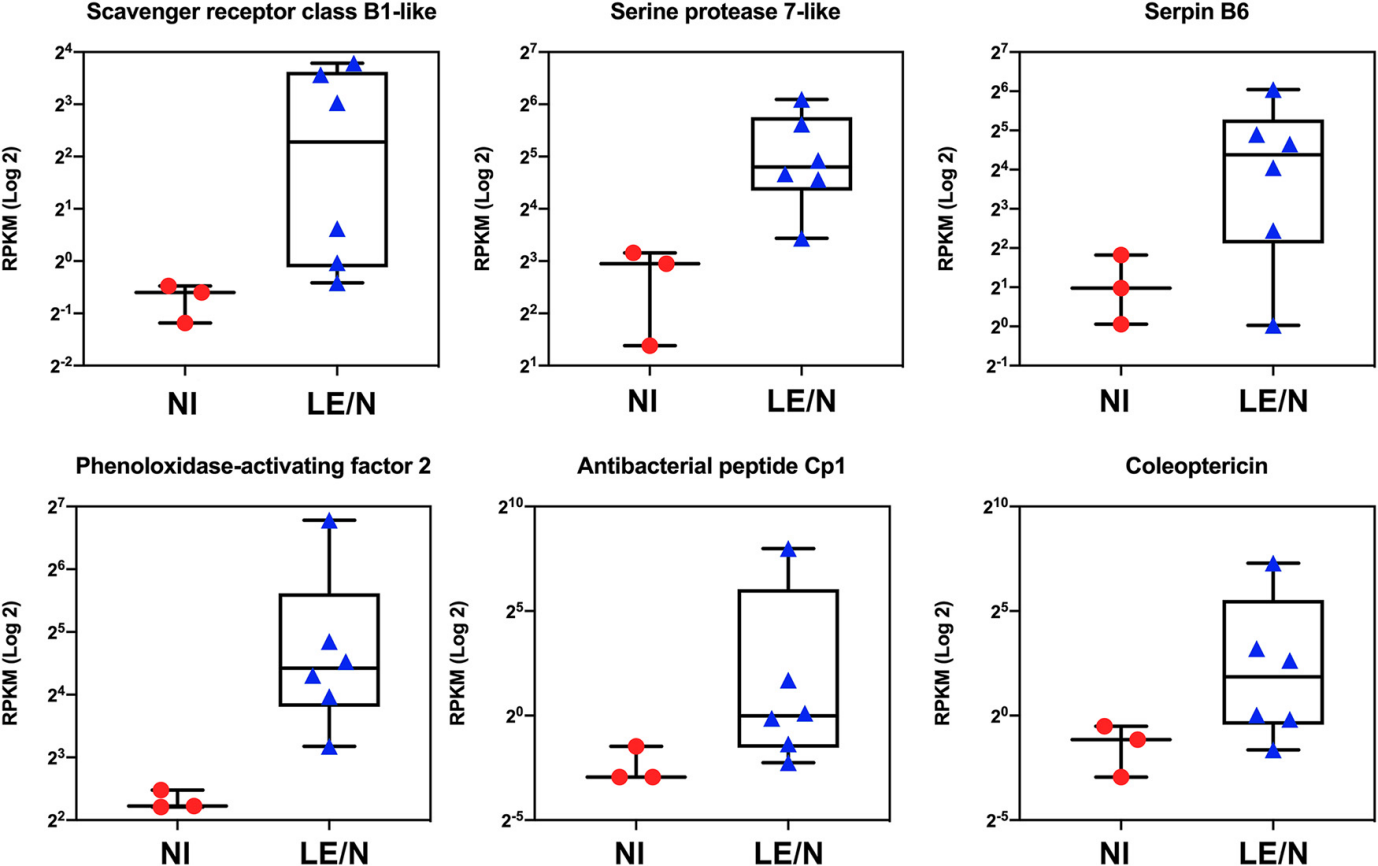
The gene expression profile of noninfected individuals of newly introduced (NI; Solomon Islands) and long-established or native-range (LE/N; Fiji, PNG, and The Philippines) populations. We detected low expression values of a few immune-related genes, such as cecropin, phenoloxidase activating factor, serpin, and coleoptericin, in the newly introduced population. More details about DEGs in newly introduced and long-established or native-range populations are available in Table S4.

We also compared the gene expression profiles of noninfected individuals among the four different geographical regions. Overall, 579 sequences were differentially expressed between insects that had recently invaded a region (Solomon Islands) and those that were well established in their location (Fiji, PNG, and the Philippines) (Table S4 and Fig. 6). Interestingly, we detected low expression values for a few immune-related genes such as cecropin, phenoloxidase-activating factor, serpin, and coleoptericin, in insects from the Solomon Islands (Fig. 6). This finding suggests that this population keeps the expression of immune-related genes at low levels, possibly to minimize fitness costs associated with a hyper-immune system (65). These findings also explain the profound induction of AMPs in the recently established population after exposure to virus. The exotic population of *O. rhinoceros* in the newly invaded country (the Solomon Islands), which has not been exposed to OrNV for a prolonged period of time, displays a strong immune response compared to a population that has been exposed to the pathogen for longer. If we accept that intensifying several immune responses is costly for an invasive *O. rhinoceros* population and deteriorates their fitness, maintenance of the immune system at low levels should be preferred in this geographical population.

### Conclusion.

Our comparative analysis of OrNV-infected and noninfected *O. rhinoceros* revealed that specific sets of genes were induced by viral infection in the beetles. This induction was much stronger in *O. rhinoceros* individuals collected from the Solomon Islands, a newly invaded country, than in individuals collected from within the beetle’s native range (the Philippines) or from longer-established populations in its exotic range (Fiji and PNG). This suggests a potential role for these induced genes in the survival of individual insects after exposure to the virus. Although this phenomenon can be due to host-related characteristics, variation in virus virulence may also drive divergence in the immune responses detected. Individual *O. rhinoceros* from the Philippines and Solomon Islands that were tested in this study all belonged to the CRB-G mitochondrial lineage, but the country-specific responses of the beetles to OrNV infection were very different, providing additional evidence (see reference 1) that this mitochondrial sequence is not an appropriate marker for the response of *O. rhinoceros* to OrNV infection. Further analyses of more individuals from other newly invaded countries are now required to determine if the phenotypic response of beetles from the Solomon Islands can be associated with the capacity to invade and establish in new areas.

## MATERIALS AND METHODS

### Insect collection and RNA extraction.

Adult female coconut rhinoceros beetles (*O. rhinoceros*) were collected from the Philippines (Los Baños) and three different South Pacific countries, Fiji (Sigatoka, Viti Levu), Papua New Guinea (Kimbe, New Britain), and the Solomon Islands (Honiara, Guadalcanal) using traps baited with aggregation pheromone (Oryctalure, P046-Lure; ChemTica Internacional, S. A., Heredia, Costa Rica) from June to October 2019.

This work was conducted under the Australian Centre for International Agricultural Research (ACIAR) project HORT/2016/185. Beetles were collected by project partners at the Ministry of Agriculture in Suva (Fiji) and the Ministry of Agriculture and Livestock in Honiara (Solomon Islands) and by project collaborators at the Papua New Guinea Oil Palm Research Association (New Britain, PNG) and The National Crop Protection Center in Laguna (The Philippines).

Individual female insects were disinfected by soaking in 75% ethanol for 30 s and then rinsing in phosphate-buffered saline (PBS) before their midgut tissues were dissected, removed, and preserved in an RNA stabilization reagent (RNAlater; Qiagen). Preserved specimens were shipped to the University of Queensland, Brisbane, Australia, for further analysis. The preserved gut tissues were kept at −80°C upon arrival. For examination of virus infection status and mitochondrial haplotype, DNA was extracted from fat bodies and gut fragments using the Qiagen blood and tissue DNA extraction kit according to the manufacturer’s instructions.

The presence of OrNV in insects was confirmed by successful amplification of a 945-bp product using the OrV15 primers that target the *OrNV-gp054* gene (66) and by Sanger sequencing of each PCR product. For confirmation of the mitochondrial haplotype, a small fragment of *CoxI* gene was amplified in these individuals with the primers LCO1490 and HCO2198 (67). PCR products were sequenced bi-directionally using an ABI3730 genetic analyzer (Applied Biosystems) at Macrogen, Inc., Seoul, South Korea.

The midgut from OrNV-positive individuals was further dissected and transferred to Qiazol lysis reagent for RNA extraction according to the manufacturer’s instructions (Qiagen; catalog no. 79306). The RNA samples were treated with DNase I for 1 h at 37°C, and then their concentrations were measured using a spectrophotometer, and integrity was ensured through analysis of RNA on a 1% (wt/vol) agarose gel. After checking the RNA quality, total RNA samples were submitted to Novogene Genomics Singapore Pte. Ltd. for RNA sequencing (RNA-Seq). The PCR-based cDNA libraries were prepared using NEBNext Ultra RNA library prep kit and were sequenced using Novaseq6000 (PE150) technology with the insert size between 250 and 300 bp.

### RNA sequencing and bioinformatic analysis.

CLC Genomics Workbench version 21.0.3 was used for bioinformatics analyses. All libraries were trimmed from any vector or adapter sequences remaining. Low-quality reads (quality score below 0.05) and reads with more than two ambiguous nucleotides were discarded. We used a *de novo* assembly approach with a kmer size of 45, bubble size of 50, and minimum contig length of 500 bp to process these data. The contigs were corrected by mapping all reads against the assembled sequences (minimum length fraction, 0.9; maximum mismatches, 2). The corrected contigs arising from the *de novo* assembly were used as a reference set of genes for transcriptome analysis. Short-read sequence data from each library were separately mapped against the reference set of assembled transcripts using the CLC Genome Workbench RNA-Seq function (minimum length fraction, 0.9; maximum mismatches, 2; insertion cost, 3; deletion cost, 3) on a non-strand-specific option with a maximum of 10 hits for a read.

Expression values for all *O. rhinoceros* genes were calculated as the total mapped read count and then normalized to reads per kilobase of transcript per million mapped reads (RPKM). The gene expression profile of each individual sample was created based on calculated RPKM values. The Wald test in CLC Genomic Workbench was used to compare each sample against its control group (infected versus noninfected) for each geographical population. We considered genes with more than 2-fold changes and a false-discovery rate (FDR) of less than 0.05 to be significantly modulated genes.

All differentially expressed genes were uploaded to the Blast2GO server for functional annotation and GO analysis. We used BLAST (nucleotide [nt] and nr databases), enzyme classification codes (EC), and InterProScan algorithms (68) to reveal the GO terms of differentially expressed sequences. We also used eggNOG version 4.5 to run pairwise orthology prediction on the Cluster of Orthologous Groups (69). More abundant terms were computed for each category of molecular function, biological process, and cellular components. Blast2GO integrated the FatiGO package for statistical assessment, and this package uses Fisher’s exact test.

To identify the mitochondrial haplotypes of each individual, we mapped the clean reads to the *O. rhinoceros* mitochondrial genome (GenBank accession no. MT457815.1) and the consensus sequences that have been generated for each library (70). For the construction of the maximum-likelihood phylogenetic tree of host mitochondrial DNA, we selected a nucleotide substitution model based on the consensus outcome of the hierarchical likelihood ratio test (hLRT), Bayesian information criterion (BIC), and Akaike information criterion (AIC). The general time reversible (GTR) +G rate variation (4 categories) and +T topology variation were selected from all tests. The maximum-likelihood phylogeny was then constructed using CLC Genomics Workbench with a neighbor joining starting tree construction method under the nucleotide-substitution models mentioned above with 1,000 bootstraps.

### Data availability.

The RNA-Seq data have been deposited in the National Centre for Biotechnology Information’s (NCBI’s) Gene Expression Omnibus (GEO) and are accessible through GEO series accession no. GSE152658.
