# Supplementary material for: Diverse Host Immune Responses of Different Geographical Populations of the Coconut Rhinoceros Beetle to Oryctes Rhinoceros Nudivirus (OrNV) Infection
Source: Microbiol Spectr. 2021 Sep 15;9(2):e00686-21. doi: 10.1128/Spectrum.00686-21 (PMC8557903; doi:10.1128/Spectrum.00686-21)
Supplement: SUPPLEMENTAL FILE 5 — Supplemental material. Download SPECTRUM00686-21_Supp_5_seq13.pdf, PDF file, 0.2 MB [file spectrum00686-21_supp_5_seq13.pdf]

Supplementary Figures and Tables

Diverse host immune responses of different geographical populations of the coconut rhinoceros beetle to Oryctes rhinoceros nudivirus (OrNV) infection

Kayvan Etebari[1*], Maria Gharuka[2], Sassan Asgari[1] and Michael J. Furlong[1]

1- School of Biological Sciences, The University of Queensland, Brisbane, Queensland 4072, Australia.
2- Research Division, Ministry of Agriculture and Livestock, Honiara, Solomon Islands

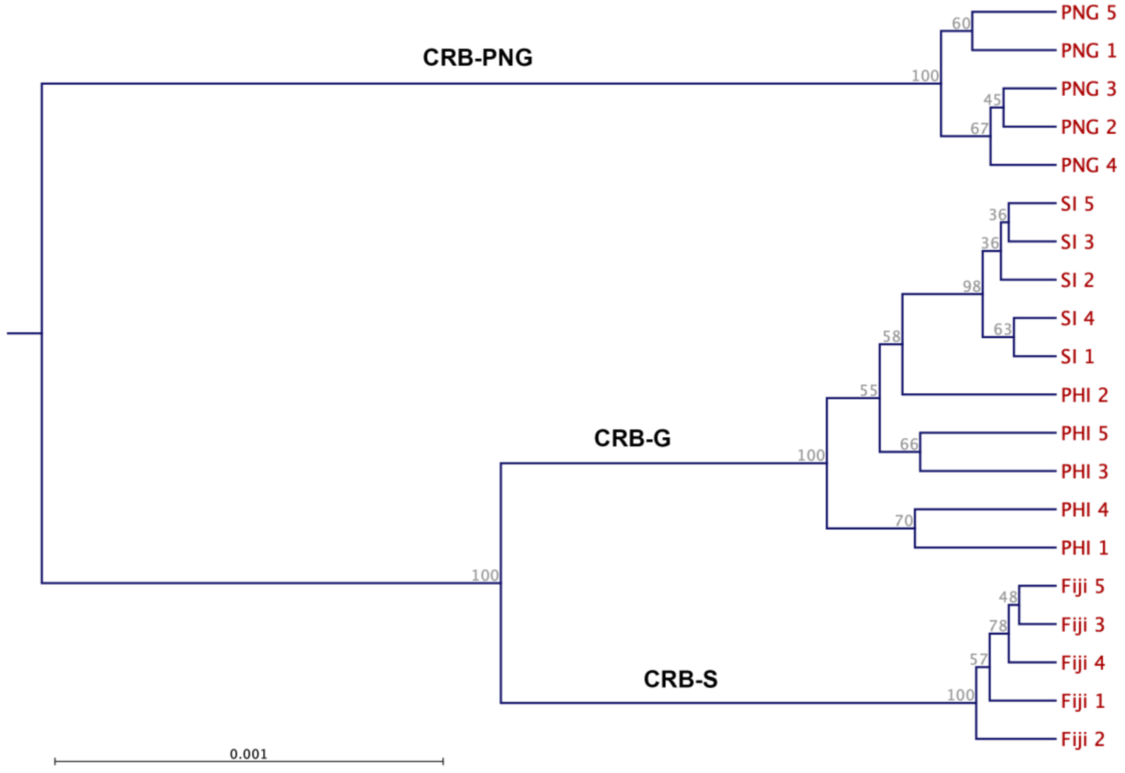

Figure S1. **The Maximum-Likelihood phylogeny of *O. rhinoceros* based on the complete mitochondrial genome.** All wild-caught individual *O. rhinoceros* classified under three major previously known haplotype group. Individuals collected from The Philippines and Solomon Islands are grouped under same group and known as CRB-G, while samples from Fiji and PNG belongs to the CRB-S and CRB-PNG haplotype groups respectively. The tree was constructed under the nucleotide-substitution model GTR+G+T with 1,000 bootstraps.

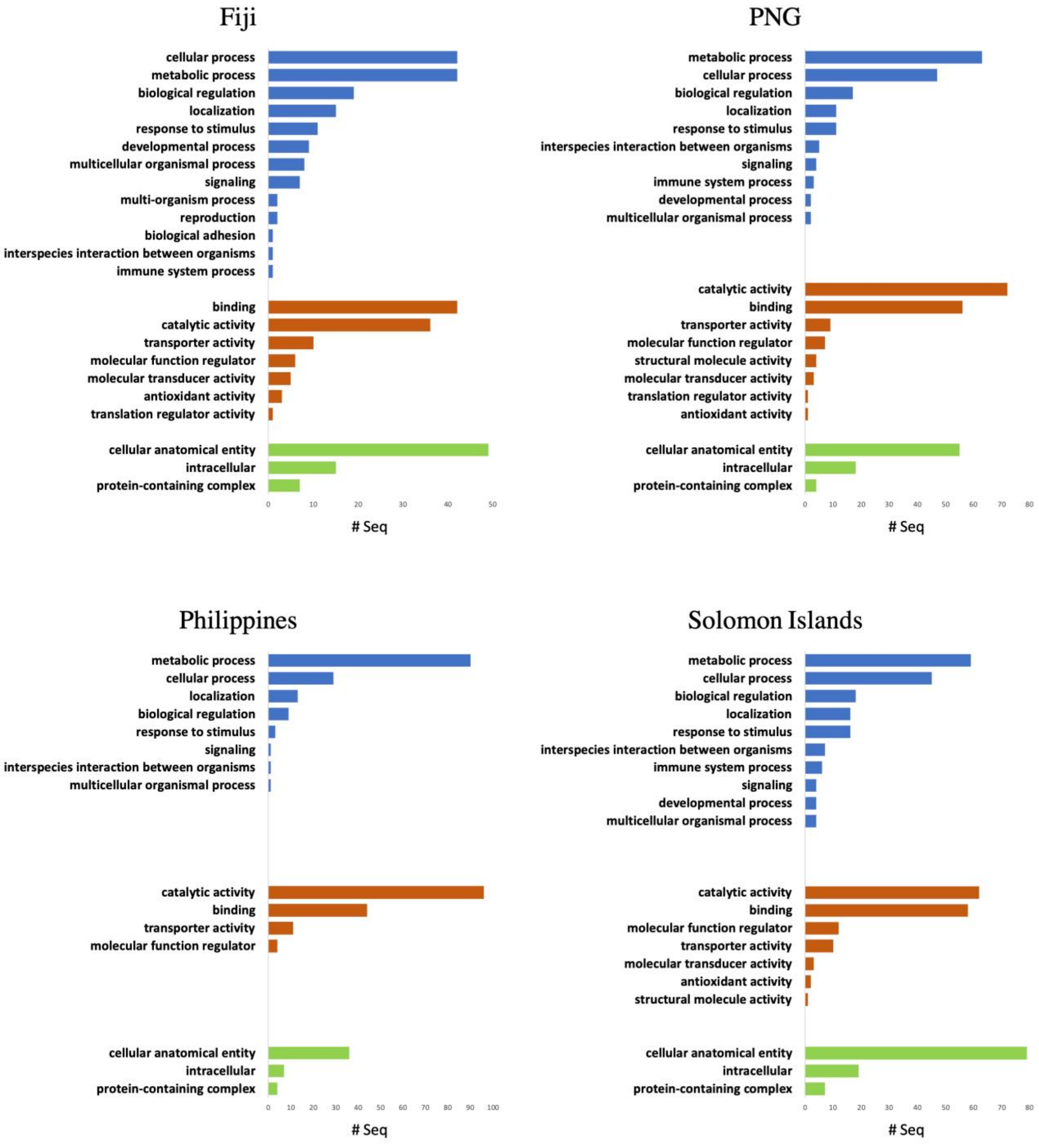

Figure S2. The Gene Ontology term enrichment analysis of differentially expressed transcript in response to OrNV infection in four geographical populations of *O. rhinoceros*. Blue colour: Biological process; brown colour: Molecular function; green colour: Cellular component. (at level of 2 GO terms)

Table S1

The top 200 highly expressed *O. rhinoceros* sequences.

Table S2

Differential gene expression profile of *O. rhinoceros* in response to OrNV infection regardless of their geographical region.

Table S3

Differential gene expression profile of *O. rhinoceros* in response to OrNV infection in each geographical population.

Table S4

The gene expression profile of non-infected individuals of newly introduced (NI: Solomon Islands) and long established or native range (LE/N: Fiji, PNG, and The Philippines) populations.
